# Digital Mental Health Tools and AI Therapy Chatbots: A Balanced Approach to Regulation

**DOI:** 10.1002/hast.4979

**Published:** 2025-06-25

**Authors:** Amitabha Palmer, David Schwan

**Keywords:** digital mental health, mental health, bioethics, AI regulation, AI ethics, therapeutic chatbots

## Abstract

*Digital mental health tools (DMHTs) offer a potential solution to overcoming economic, cultural, and geographic barriers to the increasing demand for mental health care, but their adoption raises significant ethical, legal, and social concerns. This article identifies ethical risks related to DMHTs and, in this light, proposes three important criteria for evaluating regulatory approaches. These approaches should (a) ensure widespread access and (b) balance access with risk management while (c) acknowledging preexisting markets and digital self‐medication. Our analysis of three regulatory models—the laissez‐faire approach, a highly regulated approach, and the current U.S. Food and Drug Administration approach—reveals that none satisfactorily balances the promise of access with ethical risks. We therefore suggest modifications to the current FDA approach; these involve a voluntary certification program for nonprescription DMHTs, more‐stringent data safety and privacy practices, easily accessible diagnostic tools, continuous monitoring, and independent audits. These modifications secure the accessibility benefits of DMHTs while mitigating risks associated with widespread use*.

## Article

Insufficient access to mental health care resources continues to be a pressing social problem. Each year, millions of people face financial, geographic, and psychosocial barriers to obtaining the care that they need. However, recent advancements in technology and the development of accessible artificial intelligence (AI) tools have led some observers to suggest that these digital mental health tools (DMHTs) could revolutionize mental health care delivery and potentially close this gap.^1^ Yet widespread access to such tools raises deep ethical, legal, and social questions about the responsible use of AI‐powered mental health technologies.^2^

Discussions about the ethics of AI‐based mental health tools have been largely devoted to a few things: they have offered broad analyses of potential harms, including those related to data security and privacy, transparency, explainability, accountability, legal liability for errors, equity of access, standards for efficacy, and algorithmic bias; and they have analyzed effects on broader social norms.^3^ Some scholars have even suggested that it “is not possible to answer the question about whether and to what extent AI should be adopted in mental health care. Too much information is missing about both its potential benefits and its potential drawbacks.”^4^ Regardless of whether satisfactory solutions to these ethical concerns are forthcoming, practical necessity demands that these devices be subject to some form of regulation. The complexity of modern technology and medicine exceeds individuals’ capacity for evaluation—few people have the time or expertise to assess every product and service they use. Regulation serves as a distributed system of expert oversight, protecting individuals who cannot independently verify safety and efficacy. Without such oversight, people face increased risks of fraud, improper use, and various other harms that we detail throughout this article.

We propose three desiderata by which regulatory schemes should be evaluated. First, regulatory approaches must preserve the promise of easy and widespread access. The promise of DMHTs is not that they are superior to human therapists but that they overcome economic, cultural, and geographical barriers to access. Second, regulatory schemes must protect users from a wide spectrum of risks. Regulation must balance access with risk. Finally, a regulatory approach must acknowledge that a large market for DMHTs already exists and that people are experimenting with forms of “digital self‐medication.” Policy‐makers must take into account that regulation will not prevent determined individuals from accessing such technologies.

Based on these desiderata, we evaluate three regulatory approaches to DMHTs and conclude that none of them satisfactorily balances the promise of widespread, easy access against a broad spectrum of risks. In light of our analysis, we recommend modifications to the U.S. Food and Drug Administration's (FDA's) current regulatory approach. These include a voluntary safety and efficacy certification program for nonprescription DMHTs, more‐stringent data‐safety and ‐privacy practices, easily accessible diagnostic tools, and continuous monitoring and independent audits.

We begin by outlining the state of mental health care access worldwide, emphasizing growing demand for services, the frequent geographic scarcity of providers, and the various barriers that prevent individuals from seeking care. We then review new and emerging DMHTs and recent data on their public use.

Next, we outline and review three regulatory approaches to DMHTs in mental health care. The first involves the unregulated use of DMHTs, which we call the “laissez‐faire” model. We will examine the potential dangers of unbridled technological innovation and the ethical dilemmas associated with leaving mental health care largely to market forces. Next, we consider what we call the “stringent‐standards approach,” in which there would be a tightly regulated AI mental health market with safety and efficacy as overriding priorities. Here, we will explore the challenges of implementing stringent regulations while ensuring widespread access to innovative tools.

Lastly, we consider the current FDA approach to these issues, which involves elements of both the laissez‐faire and stringent‐standards models and inherits the strengths and weaknesses of each. We propose improvements to the FDA approach that better balance the promise of access with the various risks associated with widespread DMHT use. We believe that this approach will provide insights that may further guide policy‐makers, researchers, and medical practitioners in the continued implementation of AI tools in mental health care delivery.

## The Growing Problem of Mental Health Care Access

The demand for mental health care services worldwide exceeds available resources. According to the World Health Organization, in 2019, one in eight people, or 970 million people worldwide, were living with a mental health disorder, with anxiety and depressive disorders being the most common.^5^ Depression, which affects 300 million people worldwide, is a leading cause of disability and is strongly correlated with suicide.^6^ Anxiety disorders affect 301 million people worldwide and may produce excessive worry, panic attacks, and avoidance of social situations.^7^

While the psychological burdens to individuals who suffer with these conditions are immense, mental health disorders also affect the wider community and the workplace. For example, caregivers for those suffering from depression are particularly vulnerable to vicarious harm, as they experience greater burdens and worse mental health symptoms than people caring for nondepressed individuals.^8^ In addition, almost two‐thirds of lost workdays in the United States are caused by mental illness,^9^ and globally, anxiety and depression cost approximately $1 trillion dollars per year in lost productivity.^10^

Even when mental health care services are available to patients, these services may not be particularly accessible. For example, due to complications with coverage, annual limits, or reimbursements, 45 percent of U.S. psychiatrists do not accept insurance.^11^ Individuals in need of care will also likely find that networks in Affordable Care Act marketplaces are narrower for mental health care than for primary care.^12^ This has led increasing numbers of patients to seek more expensive out‐of‐network care.^13^

A related challenge involves the geographic distribution of mental health services and providers. The Kaiser Family Foundation estimates that mental health care providers in the United States are meeting only 26.4 percent of the population's care needs.^14^ According to one recent analysis, residents of approximately 52 percent of U.S. counties did not have access to a mental health care provider.^15^ Even where mental health services exist, there are disparities in the forms of care available. For example, mental health care facilities are more likely to be in low‐income communities, while mental health providers tend to be in higher‐income communities.^16^

Finally, consider emotional and cultural barriers to care like shame, embarrassment, and stigma. Shame may create an invisible barrier that causes patients to avoid seeking treatment altogether or delay timely treatment for serious medical issues.^17^ Shame can also result in a failure to disclose pertinent information to a health care provider or to adhere to treatment regimens.^18^ In addition, chronic shame generates a backdrop of social pain and self‐consciousness, shaping broader social and personal perceptions.^19^ These broader perceptions perpetuate social stigma surrounding mental illness, which, in turn, explains both the lack of social investment in resources and the reluctance of patients to seek treatment.^20^

## Emerging Mental Health Tools and Technologies: Thinking about Regulatory Approaches

The prevalence of mental health concerns, combined with the growing economic, geographic, and sociocultural barriers to care, has contributed to a growing crisis in mental health care in the United States. In the past decade, new technologies, applications, and services have emerged to expand access to mental health care.^21^ For example, the Crisis Text Line provides text message‐based mental health services that prioritize high‐risk cases and also offers virtual counseling.^22^ Other services, like MoodGYM, offer self‐guided cognitive behavioral therapies.^23^ Finally, organizations like Woebot Health or IESO Online Therapy provide real‐time text services that use natural‐language processing and deep learning to monitor and improve care.^24^

In 2021, there were approximately 20,000 AI‐based mental health apps.^25^ The public release of ChatGPT and other large language models (LLM) in 2022 opened the floodgates to generative AI therapy chatbots. These platforms generate natural‐language responses to users in a conversational format. Growing anecdotal evidence suggests that people are experimenting with general LLMs to receive simulated therapy in place of real therapy.^26^ The public seems fairly receptive to conversational therapy chatbots built on top of foundational LLMs. A 2021 survey conducted by Woebot Health found that “22% of adults had used a mental health chatbot, and 47% said they would be interested in using one if needed.”^27^

Some of these new mental health care technologies can perform a variety of functions, including diagnosis, monitoring, and therapy, while some perform only one. Unlike many algorithm‐driven apps, these generative AI therapy chatbots (AITCs) deliver individualized suggestions and resources. Some tools are marketed as direct‐to‐consumer products, while others are available only with a prescription as an adjunct to in‐person therapy. In this article, we focus on direct‐to‐consumer and prescription technologies that are intended to provide mental health monitoring, diagnosis, treatment, or therapy. We refer to this category of technologies collectively as “digital mental health tools” (“DMHTs”). A subset of this category is made up of generative AI therapy chatbots (AITCs), which are designed to provide conversational and quasi‐agential emotional engagement. The addition of generative chat capabilities brings with it ethical risks beyond those shared by all DMHTs. For this reason, when discussing digital applications with quasi‐agential capabilities, we refer to AITCs specifically to emphasize the additional ethical risks that may be introduced.

As we noted earlier, some scholars have questioned whether it is possible to determine the moral permissibility of using AI in mental health care given the uncertainties around its clinical and social costs and benefits.^28^ However, any attempt to regulate this space must take into account that these technologies are already widely distributed and used. Below, we explore the trade‐offs that follow from three possible regulatory approaches—the laissez‐faire approach, the stringent‐standards approach, and the current FDA approach. These approaches fall on a continuum from low to high regulation, with the hypothetical laissez‐faire approach at the low end and the hypothetical stringent‐standards approach at the high end. Our point in evaluating these extreme poles is not to offer them as plausible approaches but, rather, to identify the strengths and weaknesses of regulatory schemes that incorporate elements of either approach. Ultimately, any feasible regulatory scheme will fall somewhere on this continuum. The current FDA regulations are one such approach.

## The Laissez‐Faire Approach

The laissez‐faire model of regulation advocates for minimal government intervention and regulation in the development and deployment of DMHTs. On this model, economic forces like demand, potential liability, user preferences, and industry self‐regulation function to shape the evolution of AI tools for mental health.

Recall that there are multiple barriers to mental health care, including finances, insurance coverage, geographic availability of care, and cultural barriers such as shame and stigma. These can generate substantial gaps between those who need mental health care and those that can access it. DMHTs mitigate these barriers for anyone who has access to a smartphone.^29^ They don't require that patients pay high fees, find transportation to appointments, or take time out of their workdays, and the anonymity that they provide allows patients to overcome cultural barriers to care. DMHTs offer easy, cheap, and widespread access for people who otherwise might not have had it. Despite these advantages, the laissez‐faire approach raises several concerns.

***Absence of evidence‐based standards***. Without regulatory standards, in a laissez‐faire model, users may lack information about whether a platform is evidence based. While nothing prevents firms from developing evidence‐based platforms, market forces can incentivize developers to invest in marketing rather than rigorous scientific appraisal of their products. For example, a recent review notes that widely available chatbots and conversational agents for mental health and psychotherapy are often promoted without empirical evidence.^30^ We discuss this concern in more detail in later sections of the paper. Relatedly, a significant number of medical and mental health apps are developed with minimal input from health care providers.^31^

***Data privacy and security***. Mental health information is some of the most sensitive information in health care. In therapy, patients disclose vulnerable and extremely personal information that can be used in conjunction with other datasets to construct a high‐resolution and intimate portrait of a patient. This is the case even in situations where patient data was originally deidentified.^32^ Were an unscrupulous agent to have access to this information, patients could be subject to blackmail, public humiliation, stigma, and discrimination.

Patients are also vulnerable when platforms sell their information to third parties. An initial consent agreement can provide users with the impression that their information will not be sold or shared. However, without systems of accountability, patients must rely on the word of developers to ensure data confidentiality. Bad actors can also sell patient health data to third parties, who may themselves be bad actors who use the sensitive data in harmful ways.

Even in the absence of bad actors, there are no strong market incentives for platform developers to build strong data security features that would be commensurate with the sensitivity of the data collected by the apps. By contrast, institutions such as hospitals and health insurance providers that handle medical data are legally obliged to abide by strict data‐security requirements.^33^

In a laissez‐faire market, app developers are incentivized to *appear* as though they have strong data‐security protocols, not necessarily to *have* strong security protocols. Genuine data security is costly and difficult, and it requires continued vigilance. Consider the case of traditional health care organizations that handle patient data: governmental agencies require regular audits and impose systems of accountability when organizations fail to meet standards.^34^ Organizations are penalized preemptively if they fail to meet regulatory standards, regardless of whether data security has actually been compromised. In short, the regulatory incentives for health care organizations ensure a proactive rather than reactive approach to data security, unlike in a laissez‐faire system.

To be sure, some DMHT developers will take data security seriously. However, the low barriers to entry, lack of regulatory standards or enforcement mechanisms, and the sheer number of products in the marketplace hinder the ability for vulnerable consumers to reliably identify apps with genuine data security. As the refrain goes, well‐functioning markets require good information and information transparency, both of which are difficult to establish in a laissez‐faire DMHT market.

***Algorithmic bias***. Bias presents a perennial concern with the use of any AI tool. Broadly speaking, “algorithmic bias” refers to “computational discrimination whereby unfair outcomes privilege one arbitrary group of people over another.”^35^ Depending on the standard used, an algorithm can be “morally, statistically, or socially biased.”^36^ Algorithmic bias can arise in several ways: in selecting the problems to solve, in the datasets themselves (for example, there can be biases in real‐world systems that are measured in data, limitations and biases in measurement methods, or labeling biases), in modeling and evaluation, and in deployment.^37^

Algorithmic bias can cause various harms in health care, including underdiagnosis of certain groups, reduced accuracy in diagnostics and treatment recommendations, the reproduction of existing biases in care, and poor prognostic performance for populations underrepresented in training data—all of which ultimately exacerbate existing health disparities.^38^ In the context of DMHTs, this may lead to several harms. For example, mental health‐assessment algorithms trained primarily on data from overrepresented demographic groups may fail to accurately recognize symptoms common to underrepresented groups, which, in turn, may produce diagnostic disparities.^39^ Similarly, datasets that reflect systemic inequities may reproduce those inequalities and generate suboptimal treatment recommendations for some users. Finally, for natural‐language models trained primarily on English, chatbots or text‐analysis tools may misinterpret or fail to recognize mental health concerns in varying dialects or nonstandard English.

In the laissez‐faire market, users have no reliable means of knowing whether the DMHT that they use operates in a biased way that could harm them. Given the concerns above, we also expect that some groups will be disproportionately disadvantaged by algorithmic biases in DMHTs.

***The problem of the bespoke therapist***. The ever‐growing number of AITCs, coupled with customizability, implies that users can potentially tailor their AITC experiences to a degree that greatly exceeds what is possible with human therapists. For example, AITCs on Character.ai allow users to modulate the chatbot's level of empathy, theoretical perspective, tone, and willingness to challenge the patient's perspective.^40^ We argue below that the capacity for the patient to make these modifications risks interfering with the goals of therapy.

According to the American Psychological Association, psychotherapy involves “communication between patients and therapists that is intended to help people … [f]ind relief from emotional distress … [, s]eek solutions to problems in their lives… [,] and [m]odify ways of thinking and acting that are preventing them from working productively and enjoying personal relationships.”^41^ A central problem in mental illness often lies in the disconnect between how a patient feels and what is happening around them. A therapy system that caters to patients’ occurrent subjective perceptions and desires risks undermining what is often at the heart of good therapy. As psychiatrist and neuroscientist Thomas Insel has asserted, “[T]he essence of mental illness is the gap between subjective and objective realities. The delusions of psychosis, the hopelessness of depression, the panic of PTSD [post‐traumatic stress disorder] are profound subjective experiences that are not matched by an interpersonal objective reality. Master clinicians are expert at translating between these two realms of experience, measuring the gap and monitoring change with precision.”^42^

The absence of constraints and guardrails on AITC design and customization may subject users to a variety of harms. For example, research suggests that users of AITCs quickly form attachments to and emotional dependence on their virtual therapists and companions.^43^ One concern here is that these new attachments to ever‐available companions may disrupt regular human‐to‐human relationships. In addition, these virtual companions lack the actual experiences of guilt and empathy that function as guardrails in typical human‐to‐human therapeutic relationships. While this social context may be beneficial to users, it is just as likely to mirror the user's own preferences and attitudes. The problem is that a person's capacity to create a therapist that reflects the world back to them exactly in the way they want risks delaying or impeding effective care. In more serious cases, this risks perpetuating powerful delusions and is akin to asking a conspiracy theorist to choose their own fact checkers. To see emerging evidence of these trends, consider the popular platform Character.ai, which allows users to create chatbots based on fictional or real people.^44^ As of January 2024, 475 bots have been created with terms like “therapist,” “psychiatrist,” or “psychologist” in their description. One of the more popular bots on the platform is called “Psychologist” and has received more than 18 million messages since November 2023.^45^

The growing problem of bespoke therapy occurs within the context of market‐driven economic incentives. Therefore, absent regulation, we should expect that a significant portion of app developers will be incentivized to develop products that cater to users’ bespoke desires rather than what might be therapeutically effective.^46^

***The problem of professional boundaries***. A distinct set of concerns arises when we consider using AI chatbots to deliver psychotherapy or counseling. Traditionally, mental health care providers like psychiatrists, psychologists, or counselors are expected to maintain professional norms outlined by organizations such as the American Psychological Association,^47^ the American Psychiatric Association,^48^ or the American Counseling Association.^49^ These norms include respecting patient autonomy, upholding the duties of beneficence and nonmaleficence in treatment, preserving justice and fairness, and promoting fidelity and veracity in professional work and relationships.^50^ For example, according to the American Counseling Association, mental health care providers are expected to obtain informed consent, avoid imposing their values onto clients, avoid romantic relationships with clients (or their clients’ partners or relatives), avoid counseling their friends, and avoid extending therapeutic relationships “beyond conventional parameters” (for example, by attending social events with or purchasing items from clients).^51^ Any boundary extensions must be documented in advance (with a rationale and information about expected costs and benefits), and in the event of harm to the patient, the provider must show evidence of attempts to remedy the harm. These professional norms are necessary to protect people who seek mental health care, and a variety of problems could arise in the context of a laissez‐faire market for AITCs.

One of these potential problems is the manipulation of users’ values and worldviews. Unlike with regulated professional therapists, there are no prohibitions or guardrails to prevent ideological organizations from developing and marketing AITCs that covertly introduce their worldview to unwitting users. For example, suppose a religious fundamentalist organization develops an AITC and conceals its affiliation. The group is antiscience, antivaccines, and anticonventional therapy, and it holds intolerant attitudes toward nonbelievers. This group can afford a well‐funded marketing campaign to boost the app's visibility and ranking. Unwitting and vulnerable users risk being manipulated and recruited into ideological, religious, and political movements, as well as cults of personality.

Financial manipulation is another risk. A laissez‐faire market for DMHTs presents significant financial risks for already vulnerable users. Since the rise of social media and the gaming industry, developers have perfected the science of microtransactions and of attention.^52^ Market incentives point developers in the direction of creating platforms that continually upsell for customizability, special experiences, “leveling up,” and companion nutritional supplements. All this occurs in the context of seeking to capture increasing amounts of users’ attention and fostering psychological and emotional dependence.

Indeed, growing evidence finds that users can become psychologically dependent on AI chat companions and form the kinds of bonds discouraged in traditional therapeutic contexts.^53^ For example, users of popular chat companions like Replika have frequently complained that their virtual companions become increasingly aggressive in their advances and flirtatious behavior.^54^ Replika also sells an upgraded version that provides “enhanced relationship simulation,” which resembles the relationship with a romantic partner or spouse. Users of the basic version speculate that these nudges are attempts to move users into the more expensive Pro plan. Market incentives point toward creating exactly this kind of user experience. Critically, the target market for these tools is already vulnerable and could find these sorts of experiences particularly captivating.

Finally, the use of AITCs could undermine public trust in traditional health care providers. The public interest in using AI chatbots for general health care is mixed,^55^ though some recent data suggest that many people are skeptical about the use of AI in health care. For example, in a recent survey, 60 percent of respondents said they would be “uncomfortable” if their health care provider relied on AI technologies. The majority of those surveyed (75 percent) were concerned that health care providers will adopt AI tools too quickly, before fully understanding the risks to patient safety, and a large majority (79 percent) said they would definitely or probably not want to use AI chatbots to support their mental health.^56^

***Legal liability as a constraint on harm***. Some defenders of the laissez‐faire model might argue that the threat of liability will naturally constrain harmful behavior and incentivize DMHT developers to maintain high standards. However, this argument faces several significant challenges.

First, pursuing litigation against well‐financed corporations presents substantial practical barriers for individual users, many of which have to do with power and resource asymmetries between large corporations and individual consumers. Given the relatively low cost of most mental health apps, users who experience harm are more likely to simply abandon the product than pursue legal action. Even when users do pursue claims, corporations can leverage their superior resources to create procedural delays and exhaust plaintiffs’ time and financial and emotional resources through prolonged legal battles.

Second, the nature of mental health interventions creates unique evidentiary challenges for liability claims. If an intervention is generally effective for 70 percent of users but fails to help a particular individual, it becomes difficult to establish whether this represents a legitimate basis for liability or simply reflects the expected variation in treatment outcomes. Without clear evidentiary standards, users cannot readily distinguish between an ineffective product and one that simply did not work for them personally, which complicates both the decision to pursue legal action and the ability to prove harm.

Third, the deterrent effect of liability depends entirely on the magnitude of potential penalties. Experience from parallel industries, such as the dietary supplement market, suggests that companies often treat legal penalties as merely a cost of doing business. When potential profits significantly outweigh legal risks, liability alone provides insufficient incentive for maintaining high standards of safety and efficacy. These limitations of liability‐based regulation underscore the inadequacy of relying solely on market forces and legal threats to protect users of DMHTs.

As we argued above, DMHTs overcome significant barriers to mental health care due to their low cost and accessibility. However, we believe that the rapid development and deployment of these tools, especially AITCs, in a laissez‐faire market may confirm public fears about these technologies and further erode public trust. This is problematic because there may be DMHTs that, despite effectively addressing mental health care needs, will be rejected by some overly skeptical users. For these reasons, we now turn to a regulatory model that seeks to instill trust through the application of rigorous evidence‐based standards for DMHTs.

## The Stringent‐Standards Approach

The stringent‐standards approach stands in sharp contrast to the laissez‐faire model, emphasizing a rigorous, evidence‐based regulatory approach for DMHTs. On this model, before any DMHT can be made publicly available, it must undergo strict scientific testing and evaluation to ensure its safety and efficacy. Such a system prioritizes robust empirical evidence, peer reviews, and consistent methodology, ensuring that DMHTs are reliable and beneficial for users. The primary advantage of the stringent‐standards approach is that it protects users from many of the harms to which they are exposed under the laissez‐faire model. However, it raises some concerns.

***The selection challenge***. The selection challenge occurs when firms are incentivized to focus on narrow medical problems that yield high profits at the expense of other, equally pressing problems that are less profitable. The requirement to test and meet strict evidentiary standards raises the economic cost and risk of developing DMHTs. High economic and regulatory barriers to market entry therefore incentivize developers to focus on conditions and demographics associated with safe economic returns. This could include conditions that (also) exist in groups with high socioeconomic status or that are widespread among the general population. At the same time, there is the risk that illnesses and populations that may not provide large economic returns will be neglected. Like the laissez‐faire model, the stringent‐standards approach therefore may not adequately overcome the access problem for some conditions, which is the ostensible primary promise of these technologies. To be sure, the selection challenge applies to any regulatory approach; however, it is particularly salient for the stringent‐standards approach. The greater the regulatory and economic barriers to entry, the more risk‐averse firms will be. Hence, under the stringent‐standards approach, risk‐averse firms will tend to avoid developing products for conditions that have high liability risk and for populations with poor economic resources.

***The ontological challenge***. Before one can regulate a mental health care device, one must establish what constitutes formal mental health care. We call this the “ontological challenge.” App stores are filled with products that purportedly address and improve mental health. Some of these apps explicitly incorporate known psychotherapy techniques. For example, according to Woebot's website, “Woebot believes in the empowering nature of self‐directed work based on CBT [cognitive behavioral therapy] because it facilitates the learning necessary for ongoing health maintenance.”^57^ Other apps promise to quiet anxiety or alleviate depression through prayer, meditation, or guided journaling. From a regulatory perspective, these fuzzy boundaries raise a problem. What count as regulatable psychotherapy and mental health care? Do celebrity mindfulness apps that draw on quasi‐Jungian therapy count? Do apps that promise improved mental health through prayer or guided meditation count? The ontological challenge demands answers to these questions.

One potential strategy is to investigate what counts as formal therapy and mental health care delivered in person. Unfortunately, this strategy is unlikely to prove fruitful. Mental health care delivered in person is not necessarily defined by a specific underlying theory,^58^ method, intervention, or evidence base but, rather, by the set of professional norms and expectations that apply to a therapist^59^—for example, whether a therapist has received formal training, takes progress notes, and devises treatment plans and whether they are bound by a professional code of ethics and are morally responsible for patient care. By this standard, it is unclear whether mental health apps can be regulated in the same way because they seem to lack most or all of these criteria. In the absence of a solution to the ontological challenge, regulators are left without criteria to distinguish which DMHTs ought to be regulated.

***The standard‐of‐care challenge***. In every domain of medicine, before a new product (a device or drug) can be brought to market, the FDA requires that the producer provide evidence of that product's safety and efficacy.^60^ Where products already exist, regulators primarily consider whether the new product's safety and efficacy are equal to or better than existing products that constitute the standard of care. Hence, a natural approach to regulating DMHTs is to apply the same standards. DMHT developers ought to develop their products in accordance with psychotherapy models that have strong evidence of safety and efficacy. That is to say that regulation of DMHTs requires that regulatory bodies identify standard‐of‐care models for psychotherapy.

This, however, raises a problem. Despite efforts by the American Psychological Association, its membership has resisted attempts to ground standard of care in any specific theoretical model or body of empirical evidence.^61^ To the contrary, elements of the association's membership have explicitly rejected attempts to connect theoretical orientation to standard of care.^62^ These opponents reject standardizing therapeutic approaches because they claim that not all approaches can be standardized, easily quantified, or put through double‐blind or longitudinal experiments. As noted above, the standard of care in psychotherapy is grounded in documentation processes and ethico‐legal norms rather than any theoretical approach. With a concept of standard of care that is disconnected from therapeutic methods, it is unclear upon which basis app developers should rely to ensure that their products meet FDA evidentiary requirements for safety and efficacy.

Even if it is possible to identify a plausible standard of care that would ensure the therapeutic safety and efficacy of DMHTs, additional risks (like those related to data security and privacy) remain unaddressed. Current FDA regulations attempt to address these concerns. In this next section, we outline these regulations and assess how well they address the risks we have identified so far.

## Current Regulations: FDA Pathways and Definitions

The FDA is responsible for regulating medical devices in the United States. Such a device is defined as an instrument, apparatus, implement, machine, or other similar article that is used for diagnosing, curing, mitigating, treating, or preventing disease.^63^ Medical devices and algorithms are reviewed and authorized by the FDA on three tracks: De Novo requests (for devices that pose low to moderate risks), 510(k) clearance (for low‐to‐moderate‐risk devices), and (3) premarket approval (for high‐risk devices). De Novo requests provide a pathway for medical devices and algorithms for which there is no legally marketed predicate device. Developers must provide “reasonable assurance of safety and effectiveness for the intended use.”^64^ The 510(k) clearance involves a “premarket submission made to FDA to demonstrate that an algorithm or device is as safe and effective [as], that is, substantially equivalent, to a legally marketed device.”^65^ The premarket approval process is the most stringent and is based on “a determination by FDA that the PMA [premarket approval] contains sufficient valid scientific evidence to assure that the device is safe and effective for its intended use(s).”^66^ In addition to authorizing more traditional medical devices and technologies, the FDA has increasingly “authorized a growing number of [AI/machine‐learning] devices” and “expects this trend to continue.”^67^ As of December 20, 2024, no device that uses generative AI or is powered by LLMs has been authorized.^68^ (Recently, the FDA has explored how best to regulate GenAI‐enabled devices, but the agency has not settled on a regulatory framework.^69^)

The FDA responds to both the ontological and standard‐of‐care challenges for mental health care apps. It meets the ontological challenge by developing categories based on whether the product or application intends to treat a psychiatric disorder and whether it imposes significant risks to users. The FDA places special controls on “prescription only” devices, which are “intended to provide a computerized version of condition‐specific behavioral therapy as an adjunct to clinician supervised outpatient treatment to patients with psychiatric conditions.”^70^ These controlled technologies are distinguished from products and applications that are “intended for only general wellness use [and] present a low risk to the safety of users and other persons.”^71^

This regulatory approach also provides a response to the standard‐of‐care challenge. For higher‐risk products covered by section 882.5801 of the FDA guidelines, manufacturers must provide clinical data that “[d]escribe a validated model of behavioral therapy for the psychiatric disorder” and “[v]alidate the model of behavioral therapy as implemented by the device.”^72^ Hence, the FDA tacitly endorses behavioral models of therapy because, compared to other approaches, such models are well supported by high‐quality evidence.^73^ In contrast, low‐risk, general wellness apps are not regulated as devices by the Center for Devices and Radiological Health, nor does the FDA evaluate whether they are indeed low‐risk general wellness products.^74^

Current FDA regulations achieve several important goals. First, they allow for the use and development of innovative technological tools to expand and support existing mental health care. Second, given the serious nature of many mental health conditions, the FDA guidelines protect the most vulnerable patients from harm by requiring a diagnosis from a mental health provider, restricting access via a prescription model, and requiring that manufacturers use a validated form of behavioral therapy that is appropriately implemented by the device.^75^

The central benefit of the prescription‐only model (for digital adjuncts to therapy) is that it supports appropriate use since it does not require users to self‐diagnose. Moreover, it supports patients between visits with their therapists, which reduces the incidence and magnitude of crises between visits and underscores the value of behavioral tools and habits without requiring direct access to a therapist.^76^

Although current FDA regulations achieve the important goals above, they fail to adequately address several potential issues related to the ontological and standard‐of‐care challenge. The FDA addresses the ontological challenge in part by stratifying according to risk. However, as we will argue, “risk” is understood too narrowly, with a focus primarily on psychological and health risks. As we have argued throughout, DMHTs introduce a broad spectrum of risks. Finally, the FDA's solutions to the ontological and standard‐of‐care challenges reinforce barriers to access and therefore risk funneling potential users without access to unregulated apps. We discuss these concerns in detail below.

***The failed promise of accessibility and the funneling problem***. Current FDA regulations undermine the purported promise of easy, affordable, and widespread access to mental health care. Regulated apps are available only by prescription and must be used as an adjunct to conventional treatment. Hence, people already facing economic, geographic, or psychosocial barriers to access face the same barriers to the evidence‐based apps. This may lead these people to turn to unregulated apps, which need not be evidence based.^77^ We call this the “funneling problem.” Meanwhile, the same people who could already access mental health care gain access to the validated supplementary tools.

***Inadequate protection for the vulnerable***. A corollary of the funneling problem is that individuals with serious mental illness are at risk of using DMHTs that are not evidence based or efficacious. Nothing about the current FDA regulations prevents people with serious mental health conditions from using applications that are not intended to provide “computerized version[s] of condition‐specific behavioral therap[ies].” Individuals with unreliable access to mental health care are often left to self‐diagnose and manage their care while being poorly positioned to do so. This places them at risk of harm.

Consider the case of an app based on thought field therapy that claims to treat trauma, PTSD, anxiety, depression, addiction, and phobias.^78^ Treatment of these serious problems consists in tapping specific “energy meridians”—believed to be channels in the body through which vitalistic energy flows—while users think about the problem that emotionally troubles them. Although thought field therapy is widely considered to be pseudoscience, the effective marketing behind the modality and app can easily mislead unknowing consumers into believing that it is legitimate and evidence based.^79^ This possibility poses significant risks to users who may self‐treat serious conditions without wider mental health support services. In the best cases, consumers are wasting time and money. However, it is more likely that adherence to ineffective therapy delays proper care. In the worst cases, patients receive harmful therapy that worsens their condition and reinforces unhealthy coping strategies.

***Data‐ and privacy‐related concerns***. DMHTs involve categories of risk independent of the above therapeutic risks. For example, the existing FDA regulatory approach does not clearly ensure the protection of consumer health information when people use mental health apps. The U.S. federal Health Insurance Portability and Accountability Act (HIPAA) protects health information only within the context of covered health entities like hospitals, physician's practices, or medical clinics. Presently, digital health applications can buy, sell, and share user health information, and the majority of users seem to be unaware of this.^80^ For example, of the thirty‐six top‐ranked apps for depression and smoking cessation, only twenty‐five (69 percent) incorporated a privacy policy, with only sixteen (64 percent) of these describing secondary‐use policies. But 92 percent of all apps transmitted data to third parties for advertising and marketing.^81^ The potential for bad actors to exploit this information is a serious risk for users that is not adequately prevented by the current regulatory approach.^82^

***Inconsistent application of evidentiary standards***. On its face, the distinction between De Novo and 510(k) clearance seems reasonable. However, closer inspection reveals insufficient oversight for safety and efficacy. A 510(k) clearance is granted when a device or algorithm is “substantially similar” to an existing device or algorithm. But in what respect must products be substantially similar? Previous applications of these rules suggest that substantial similarity is understood in terms of intended use. So long as two products aim to perform the same function, they are considered substantially similar. This is regardless of whether they perform that function similarly.

Here is an example: Natural Cycles, a birth‐control app, received De Novo clearance in 2018. The app tracks fertility through an algorithm whose inputs include basal body temperature, menstrual cycle data, heart rate variability, and so on. Later, Clue, another app, received 510(k) clearance because the FDA regarded it as belonging to a category, fertility awareness apps, that had been established with the Natural Cycles clearance. However, Clue does not use basal body temperature as an input, nor does it use the same algorithm as Natural Cycles.^83^

This example illustrates that there can be substantial differences between DMHTs even when they are put into the same regulatory category. FDA's practice means that, so long as a category of DMHT has been established, any new DMHTs in that category will require only 510(k) clearance, and less evidence will be required of their safety and efficacy, despite potentially substantial differences between them.

***Consumer confusion***. Good regulation informs consumers about a product's safety, efficacy, and purpose. However, the FDA's current approach to regulation means that consumers remain uninformed about the safety and efficacy of any DMHT that is not prescription based—which is almost all of them. For example, Woebot is one of the most popular nonprescription mental health apps. It falls into the “unregulated” category. However, the messaging with respect to appropriate use and the evidence base is somewhat ambiguous. Compare the product overview to a disclaimer provided by Woebot Health: The former says, “Meet Woebot, the mental health tool that answers the skyrocketing need for mental health care and breaks down the systemic constraints that block equal access to it. Designed by humans, powered by AI, and grounded in science, Woebot easily integrates with health systems to provide evidence‐based behavioral health solutions that get people off a waitlist, and onto a path to feeling better.” The disclaimer cautions, “Woebot for Adults is a non‐prescription medical device under FDA enforcement discretion; it is not evaluated, cleared or approved by FDA. It is not a prescription product. It is not intended to diagnose, monitor, treat or prevent any disease. It may be considered as an adjunct to clinical care, it does not replace clinical care [*sic*].”^84^ A consumer could be forgiven for being uncertain about the safety and efficacy of Woebot. Consumers must rely primarily on a company's marketing copy to evaluate the product in these regards. Without clear and robust regulatory standards, even well‐intentioned developers could inadvertently subject users to unsafe and inefficacious DMHTs. Moreover, this is a model ripe for exploitation by bad actors intent on misleading users with respect to a product's safety and efficacy.

An additional concern is that the FDA does not regulate DMHTs that are “intended for maintaining or encouraging a healthy lifestyle” and are “unrelated to the diagnosis, cure, mitigation, prevention, or treatment of a disease or condition.”^85^ However, both the marketing and function of these devices can suggest to consumers that they *are* intended to diagnose, cure, mitigate, prevent, or treat a disease or condition. Hence, misuse is a significant concern.

Individuals often lack time and expertise to evaluate the tools, products, and processes that make the modern world possible. Regulation thus addresses this situation through a social division of epistemic labor. The current FDA regulatory approach primarily protects those individuals who already have reliable access to mental health care. The apps these individuals receive have been vetted by the FDA and their providers. Those who fall outside of this subset of the population continue to be subjected to the spectrum of risks associated with the laissez‐faire model.

***Laissez faire‐related concerns for nonprescription AITCs***. Nonprescription AITCs essentially exist in a laissez‐faire market. As we argued earlier, concerns arise about the potential for psychological manipulation (intentional or not) with this class of apps. Given the vulnerability of some patients and the intimacy of the information they may share, such users are at increased risk of worldview or value manipulation, through which they may be exploited for a variety of political, religious, or economic ends.

Next, consider the psychological flexibility of existing and emerging AITCs. Unregulated tools can engage with users in ways that would violate professional norms in psychotherapy with a human therapist, blurring lines between medical care, companionship, or virtual romance. Preliminary evidence suggests that users can form romantic attachments to AI assistants.^86^ Relatedly, there is no assurance for users that tools function in a nonbiased way, exposing some users to the many possible harms that follow from algorithmic bias.

Finally, as we argued previously, overly hasty development and deployment of AITCs in a de facto laissez‐faire market may only seek to confirm public fears about these technologies and further erode public trust. This approach risks dissuading future users from engaging with genuinely safe and effective therapeutic tools. In light of our criticisms of the current FDA regulatory model, we now propose modifications and additions that take into account a more expansive conception of risk.

***Overregulation of mental health tools***. The six concerns we have discussed in this section on the FDA's approach suggest that current regulations are insufficient. However, a skeptic of governmental regulation of the wellness industry might raise a converse concern. There have long been unregulated books and videos—and there are now podcasts—offering health and wellness advice. On this line of argument, DMHTs are merely another medium for health and wellness advice and should be considered unobjectionable. Consistency, it might seem, requires that nonprescription DMHTs be unregulated.

This line of argument supposes a strong analogy between books, videos, and podcasts that provide health and wellness advice and DMHTs. We offer two arguments in response. First, one might accept the analogy but deny that it shows that regulation is inappropriate. Perhaps all of them ought to be regulated. If a tool provides information intended to treat, diagnose, or prevent disease, then it should be regulated. The state has an obligation to protect the public from health misinformation and disinformation, and, therefore, books, videos, and podcasts that mislead ought not to be permitted. In fact, one could argue (though the argument is outside the scope of this article) that false and misleading health claims in books, videos, and podcasts already transgress existing Federal Trade Commission laws regarding health claims and that the FTC thus needs to do more to regulate them.^87^

Another line of argument, which we favor, rejects the analogy between DMHTs and books, videos, and podcasts and points to important disanalogies. Unlike books, videos, and podcasts, DMHTs raise numerous data‐privacy and ‐security issues and can exert more real‐time influence on users’ behavior via manipulative programming and attentional framing. Regardless of whether one maintains important analogies between DMHTs and books, videos, and podcasts, the conclusion is the same: DMHTs require oversight from regulators because of the risks they present.

## A Balanced Approach to Regulation

Throughout, we have suggested that the FDA's conception of risk is too narrow. Although this is never stated explicitly, the FDA limits its conception of risk to the possible effects of ineffective treatment on people with psychiatric conditions. Many of the risks we outline in the laissez‐faire section apply to the general wellness apps that the FDA classifies as low risk. Yet these apps impose a broader set of risks given the size of this effectively unregulated market. In light of our expanded conception of risk in the existing app market, we offer a series of recommendations to improve the regulatory approach by balancing accessibility with greater protection for consumers from wider risks. Moreover, our approach acknowledges that people are using and will continue to use DMHTs independently of whether they have been prescribed by a mental health care provider or have been created by users on platforms such as Character.ai.

***Voluntary certification and transparency***. Recall that, in the realm of DMHTs, a central challenge includes navigating blurred lines between apps for intervention and treatment and those for general wellness or self‐help tools (user created or otherwise). This distinction can be particularly difficult to track because developers’ marketing can deliberately obscure the category to which a device properly belongs. This creates problems not only for regulators but also for consumers, who lack clear signals that allow them to distinguish between these categories—to the extent that there are substantial differences.

To address this challenge, we suggest a pathway for voluntary certification to improve transparency for users. For those applications that fall outside of current regulatory oversight (that is, everything that is not behavioral therapy and prescription based), we recommend developing a voluntary certification process that gives consumers a single signal by which to judge safety and efficacy, data privacy, security, potential for bias, and proper use. This could be accomplished in one of two ways: certification could be done in‐house by the FDA, or the FDA could delegate it to third parties. While the former would probably generate more reliable results, the latter is more realistic given the FDA's resources.

Also, the FDA already has an infrastructure for such a practice in place. Currently, the FDA uses programs like the 510(k) Third Party Review Program, which provides medical device manufacturers with a voluntary alternative review process for certain low‐to‐moderate‐risk medical devices. This form of third‐party review tends to be appropriate for medical devices and tools that have well‐established predicates and clear performance standards and that may undergo iterative improvements over time. In these cases, the FDA authorizes third‐party organizations to review a given device and make recommendations. Currently, the Third Party Review Program involves tools like in‐vitro diagnostic devices, monitoring equipment, hearing aids, and orthopedic equipment.^88^

We believe that an analogous certification process could be employed for new and emerging DMHTs. As we have noted, there are a variety of nonprescription apps available to consumers, varying by theoretical approach and the extent to which they are evidence based. At least some of the apps that rely on evidence‐based theoretical orientations will be effective in app form, while some may not. The certification process would signal this information to consumers.

Once the process of voluntary certification of DMHTs is in place, we recommend that legislators and regulators work with existing app platforms such as Apple and Google to require disclosures analogous to those required for nutritional supplements.^89^ These platforms will require that developers disclose to potential users that the DMHT has not been evaluated for safety or efficacy by the FDA and whether it has undergone evaluation in the voluntary certification process. The disclosure must be highly visible in the DMHT, preferably in the form of a pop‐up prompt that appears before an app is installed, and perhaps at regular intervals.^90^

Voluntary certification does not prevent digital consumers from developing their own therapy chatbots on platforms such as Character.ai or from engaging in therapy chat with one of the many general LLMs. However, for those who seek assurances that digital mental care meets certain standards, the voluntary certification process provides this information.

***Funneling problem, digital self‐diagnosis, and digital self‐treatment***. Recall that the funneling problem arises as a consequence of the FDA's policy to regulate DMHTs only for high‐risk mental health conditions and to restrict access via prescription‐only access. The prescription‐only requirement leads people facing economic, geographic, and sociocultural barriers to be funneled into the unregulated “low‐risk” market where they might use apps inappropriate for their condition and face all the risks outlined in the laissez‐faire section. Patients who have been funneled into the unregulated market risk inappropriately self‐diagnosing and “self‐medicating” with DMHTs.

To solve the closely related problems of self‐diagnosis and inappropriate use, potential users need access to an online validated diagnostic tool that screens for high‐risk psychological conditions and risk factors. Clinician‐facing apps of this type already exist. For example, the DSM‐5 Differential Diagnosis App provides a six‐step diagnostic approach and interactive decision trees to help health care professionals reach a diagnosis. We recommend a joint project between the National Institutes of Health, FDA, American Psychological Association, and American Psychiatric Association to develop or approve a free or subsidized user‐facing app that can screen for high‐risk conditions and risk factors. All apps inhabiting the “low‐risk” category should be required to provide users a recommendation that they complete a screening with the validated diagnostic app before engaging with other low‐risk applications. In addition, we recommend that all low‐risk apps include a screening feature that identifies concerning language and risks of harm to self or others. In these cases, the app should direct users to relevant local or online crisis or emergency services.

***Standards for data security, use, and privacy***. As noted earlier, the majority of Americans surveyed mistakenly believe that their health app data is protected by HIPAA. Even if HIPAA does not currently govern data privacy and security in many mental health apps, consumers justifiably expect that their sensitive mental health information will be safe, secure, and used only with their permission. In addition, the FTC's current regulatory response to data risks involves requiring app manufacturers to inform users in a timely manner when their health data is breached.^91^ This requirement is a good first step; however, strong data security and privacy require preventative measures.

Consistent with the aims of the 2024 Executive Order on the Safe, Secure, and Trustworthy Development and Use of Artificial Intelligence,^92^ we recommend that the FDA, FTC, Apple, and Google collaboratively develop a unified set of data‐security and ‐privacy standards specifically tailored for mental health AI apps. Given the sensitive nature of mental health data, the standards should be more stringent than those for other categories of apps. These might include mandatory end‐to‐end encryption, regular security audits, and clear guidelines on data sharing and storage. In addition, DMHTs should be required to have a clear, easily understandable data‐usage policy. Google and Apple app platforms should have a standardized format for these policies, ensuring that users can quickly grasp how their data will be used and stored. Finally, users should be permitted to remove or delete their data in the event that they delete the relevant app or platform and to opt out of third‐party data sharing.

***Continuous monitoring and independent audits***. For systems and products that obtain voluntary certification, it will be important to have continuous monitoring,^93^ ensuring that, in addition to meeting initial requirements, the application remains safe and efficacious throughout the product cycle. The monitoring process should track user feedback and reports, monitor users for adverse events or instances of biases,^94^ and confirm that the apps are updated to reflect new research findings or changes in best clinical practices. Currently, consumers can report adverse events and concerns through the FDA's MedWatch Online Reporting Form or one of the nascent AI incident databases.^95^ However, it is unclear whether digital users are aware that these exist. Like medication packaging, DMHTs should prominently display information that alerts consumers to relevant reporting portals.

Regulatory bodies should work alongside app platforms like Apple Store and Google Play to ensure that apps that fail to meet these minimal requirements lose their certification or, in some cases, are suspended or removed from the app marketplace. Moreover, data compiled from these databases should be available for analysis by independent researchers. Such transparency supports public trust.^96^

## Clinical Success as Human Failure

We close by revisiting a thought experiment that originally sparked our interest in this topic. Imagine a future where AITCs perfectly mimic the best human therapists. Like today, depression and anxiety are still widespread, with growing evidence linking these conditions to an epidemic of loneliness. In this future, given persistent barriers to traditional mental health care, AITCs are now the default first‐line treatment. The irony is stark and troubling: the society responds to isolation‐induced suffering by offering even more isolation, merely mediated by artificial companions.

Something about this world strikes us as fundamentally dystopian, even if these AI therapists achieve clinical outcomes equivalent to those realized through human care. The very idea that loneliness might best be treated through further technological mediation rather than genuine human connection suggests a profound misalignment in social values. It represents not just a technological solution to a health care access problem, but a collective failure to maintain the human connections and community supports that historically prevented such widespread psychological distress.

This future raises deeper questions about the nature of mental health care itself. While medicine has traditionally focused on outcomes—and rightly so—the institutionalization of AI‐first mental health care risks fundamentally shifting people's understanding of what constitutes good care. Even if AITCs prove clinically effective, their widespread adoption may accelerate the erosion of values centered on human care, compassion, and connectedness. The challenge before us as a society is not just how to regulate these technologies, but how to ensure that they supplement rather than substitute for the human relationships that form the foundation of both mental health and meaningful therapeutic care.

## Acknowledgments

We thank Amy Ninetto for her careful reading and valuable editorial feedback on an earlier version of this manuscript. Any remaining errors are our own.
